# Selective epigenetic alterations in *RNF43* in pancreatic exocrine cells from high-fat-diet-induced obese mice; implications for pancreatic cancer

**DOI:** 10.1186/s13104-024-06757-0

**Published:** 2024-04-15

**Authors:** Tomoyuki Araki, Naofumi Miwa

**Affiliations:** 1 Department of Biochemistry, School of Medicine, Saitama Medical University, 38 Moro-hongo, Iruma-gun, 350-0495 Moroyama, Saitama, Japan; 2 Department of Physiology, School of Medicine, Saitama Medical University, 38 Moro-hongo, Iruma-gun, 350-0495 Moroyama, Saitama, Japan

**Keywords:** Epigenetics, DNA methylation, Pancreatic cells, Diet-induced obesity, Pancreatic cancer, RNF43

## Abstract

**Objective:**

Pancreatic cancer (PC) originates and progresses with genetic mutations in various oncogenes and suppressor genes, notably *KRAS*, *CDKN2A*, *TP53*, and *SMAD4*, prevalent across diverse PC cells. In addition to genetic mutations/deletions, persistent exposure to high-risk factors, including obesity, induces whole-genome scale epigenetic alterations contributing to malignancy. However, the impact of obesity on DNA methylation in the presymptomatic stage, particularly in genes prone to PC mutation, remains uncharacterized.

**Results:**

We analyzed the methylation levels of 197 loci in six genes (*KRAS*, *CDKN2A*, *TP53*, *SMAD4*, *GNAS* and *RNF43*) using Illumina Mouse Methylation BeadChip array (280 K) data from pancreatic exocrine cells obtained from high-fat-diet (HFD) induced obese mice. Results revealed no significant differences in methylation levels in loci between HFD- and normal-fat-diet (NFD)-fed mice, except for *RNF43*, a negative regulator of Wnt signaling, which showed hypermethylation in three loci. These findings indicate that, in mouse pancreatic exocrine cells, high-fat dietary obesity induced aberrant DNA methylation in *RNF43* but not in other frequently mutated PC-related genes.

**Supplementary Information:**

The online version contains supplementary material available at 10.1186/s13104-024-06757-0.

## Introduction

Pancreatic cancer (PC), ranking seventh as the leading cause of cancer-related deaths worldwide in 2018 [1], poses challenges owing to late diagnosis and poor outcomes resulting from the absence of early symptoms. This nature of PC underscores the need for therapeutic strategies as well as cancer prevention, which has attracted significant social attention, particularly given the lack of reliable screening tests for asymptomatic individuals during the early stages. Therefore, characterizing early stage PC to elucidate the mechanisms by which high-risk factor exposure transduces presymptomatic tissue into cancerous tissues is crucial.

Recognized risk factors for PC include obesity, chronic pancreatitis, diabetes, aging, male sex, and smoking [2, 3]. A possible contributor to the transition from presymptomatic pancreatic tissue to cancerous tissue is epigenetic modification: reversible but heritable changes in gene expression without amino acid mutation and known to undergo modification through lifestyle- and environmental-factors. DNA methylation, a well-known epigenetic event, regulates gene expression (*i.e.*, level of normal protein) and microRNA stability, influencing various biological processes, including development, genetic imprinting, immune response, and aging.

PC involves genetic alterations that frequently occur in genes, namely *KRAS* [4],*CDKN2A* [5], *TP53* [6–8] and *SMAD4*/*DPC4* [9], causing aberrant effector signaling by the expression of constitutively activated or inactivated mutants. Genetic alterations also induce epigenetic alterations, linking abnormal expression levels of wild-type proteins to carcinogenesis [10, 11].

Approximately 95% of PCs originate from pancreatic exocrine cells (acinar and ductal cells) with the remaining arising from pancreatic endocrine cells (Langerhans α and β cells) [12, 13]. PC progression involves transformation to invasive lesions from non-invasive lesions, which are histologically well-defined within the pancreatic ducts [14, 15], encompassing microscopic pancreatic intraepithelial neoplasias (PanINs) [16, 17] and macroscopic intraductal papillary mucinous neoplasms (IPMNs) [18, 19]. Low-grade PanIN, the most common microscopic pancreatic lesion, exhibits *KRAS* mutations in approximately 90% of cases [20]. As lesions progress in grades, additional mutations in genes, including *CDKN2A*, *TP53* and *SMAD4* emerge [21]. IPMNs are macroscopic lesions, with a 25% risk of developing into invasive PCs and often harbor mutations in genes, including *RNF43* and *GNAS* [21]. PanINs and IPMNs exhibit an increasing prevalence of epigenetic alterations with lesion grade progressions [22–24].

In our recent study, we performed an epigenome-wide analysis of isolated pancreatic exocrine cells obtained from high fat-diet (HFD)-induced obesity (DIO) mice. Obesity induces whole-genome scale abnormalities in DNA methylation in the presymptomatic stage, with enrichment in cellular processes, such as DNA repair, transcription regulation, and cell proliferation [25]. Comparing differentially methylated regions (DMRs) with those in stage IB PC, we identified three potential pathway candidates for PC development. However, the specific impact of obesity on the methylation levels of individual PC-driver genes, including* KRAS*, *CDKN2A*, *TP53* and *SMAD4*, in DIO mice remains unknown. Therefore, we aimed to investigate alterations in methylation levels across all CpG islands within these genes in DIO mice.

## Results

**Fig. 1:**
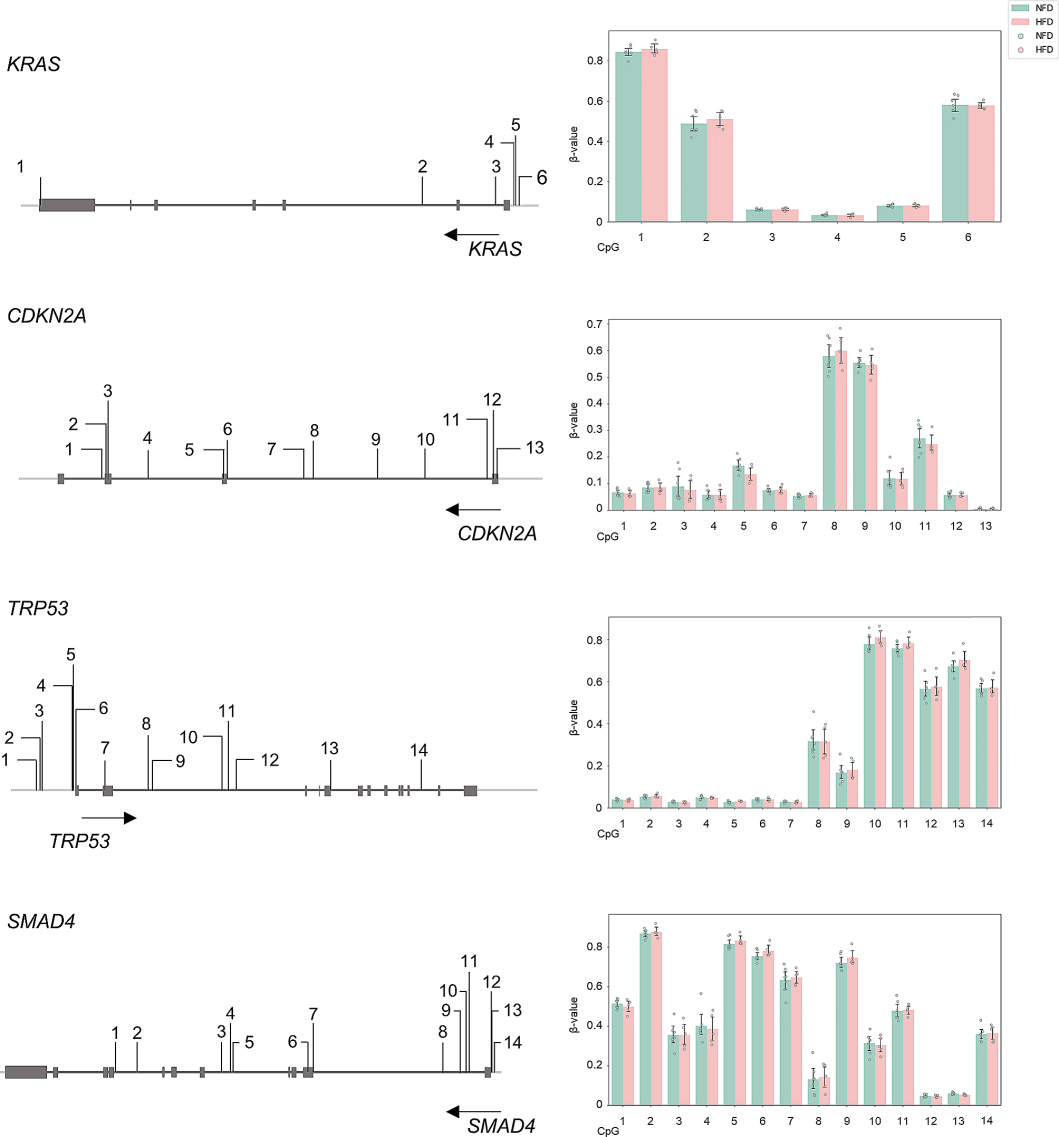
DNA methylation in key pancreatic cancer driver genes. (Left) Analyzed CpG sites in *KRAS*, *CDKN2A*, *TP53* and *SMAD4*. Untranscribed regions (pale gray bars), exons (boxes), introns (dark gray bars) and CpGs (numbers, vertical black bars) are indicated. Arrows indicate transcriptional orientation. (Right) Averaged β-values of CpGs. Methylation levels of all CpGs in *KRAS*, *CDKN2A*, *TP53* and *SMAD4* were compared between high-fat diet (HFD)- (*n* = 7) and normal-fat diet (NFD)-mice (*n* = 5), with no significant changes in averaged β-values.

Methylation in *KRAS* promoter region in mice comprised six loci: CpG#1-#6 (Fig. 1, Supplementary Table 1). Variability in methylation levels was significant across these loci, with high methylation levels in CpG#1, CpG#2 and CpG#6 (*i.e*., β-values > 0.5), and low levels in CpG#3, CpG#4 and CpG#5 (*i.e*., β-values < 0.5). HFD-induced obesity did not significantly influence methylation levels at any of these loci (Fig. 1). Methylation in the *CDKN2A* promoter region in mice comprised 13 loci: CpG#1-#13 (Fig. 1). Methylation levels in CpG#8 and CpG#9 were high, whereas those in the rest were low. HFD-induced obesity had no significant impact on methylation levels across these loci (Fig. 1). Methylation in the *TP53* promoter region comprised 14 loci: CpG#1-#14 (Fig. 1). CpGs in the 5’-region (CpG#1-#7) were almost completely unmethylated, whereas those in the 3’-region (CpG#10-#14) were hypermethylated. CpG#8 and #9, located in the middle of these two regions, were moderately methylated. Similar to *KRAS* and *CDKN2A*, HFD-induced obesity did not influence methylation levels in these 14 CpGs (Fig. 1). *SMAD4*, comprising 14 loci (CpG#1-#14), exhibited a pattern different from that of *TP53*, with hypermethylation in the 5’-region and hypomethylation in the 3’-region (Fig. 1). Overall, methylation levels in these 14 CpGs were not influenced by high-fat dietary obesity (Fig. 1). Overall, none of the methylation loci in the promoters of these four genes were influenced by HFD-induced obesity. To determine whether the methylation status of these genes underwent alterations in early stage human PC, we compared β-values from publicly available database for all methylation loci (26, 5, 10 and 14 loci for *KRAS*, *CDKN2A*, *TP53* and *SMAD4*, respectively) between early-stage PC and normal conditions. Results indicated no significant differences between normal and early-stage PC in these loci (Fig. 1).

**Fig. 2:**
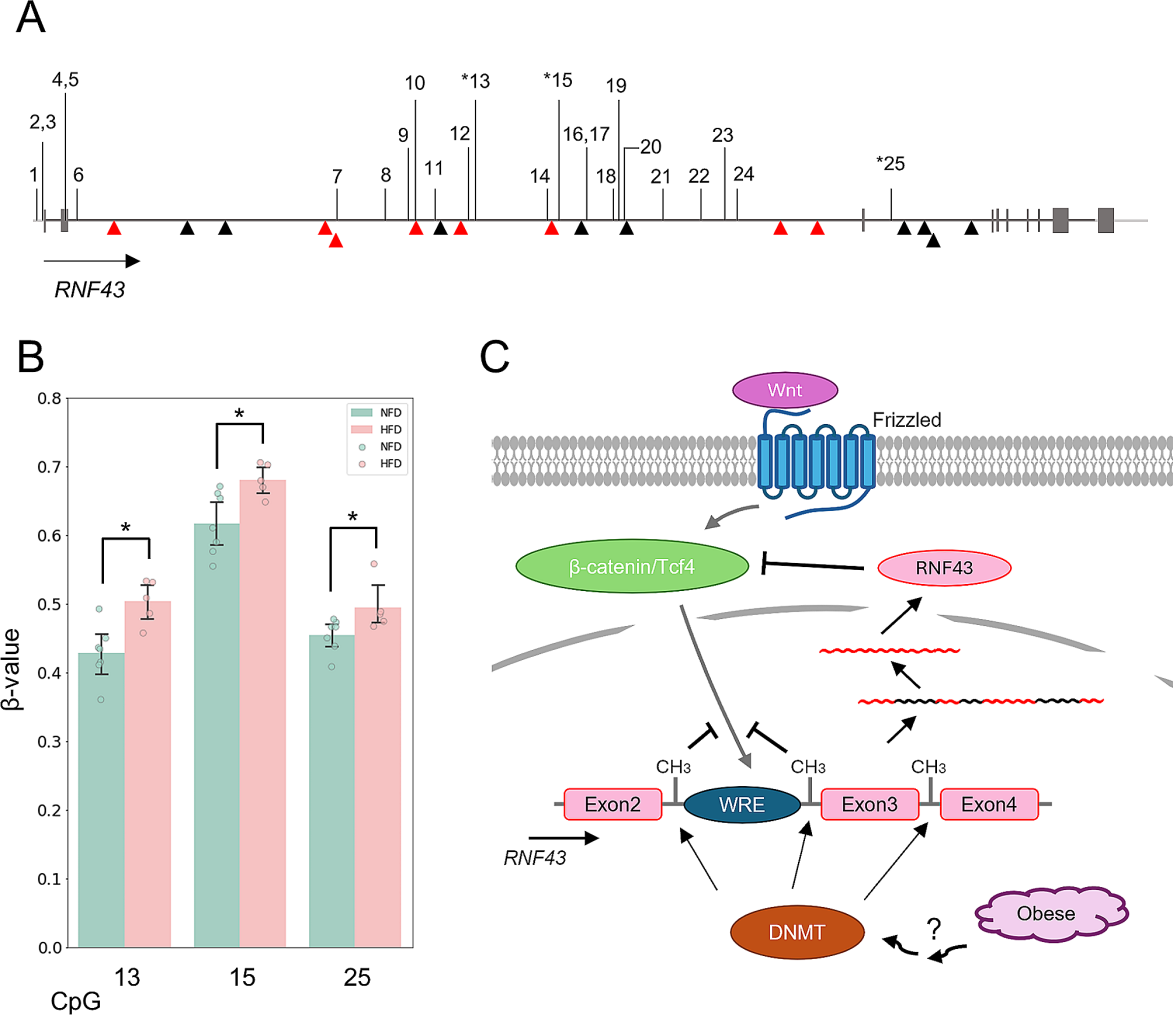
HFD-induced hypermethylation in three CpG sites of *RNF43* that negatively regulate Wnt signaling. (**A**) Gene map of *RNF43* and Wnt recognition elements (WREs). WRE1 (red triangles) and WRE2 (black triangles) are indicated. Other symbols mirror those in Fig. 1. (**B**) Averaged β-values of three CpGs in *RNF43* in HFD-and NFD-mouse pancreatic cells. Methylation levels in three CpGs (#13, #15 and #25) increased in HFD-fed mice (*n* = 7), compared with those in NFD-fed mice (*n* = 5) (*,*P* < 0.05; two-sided Student’s *t*-test). (**C**) Schematic representation of the signal transduction pathway activated by obesity in mouse pancreas. Wnt proteins bind to its cognate receptor, Frizzeled (FZD), and translocate β-catenin/TCF complex into nuclei, leading to target gene expression through binding to its translational element (WRE). RNF43 ubiquitinates FZD and induces endocytosis, downregulating the Wnt signaling.

We analyzed the methylation levels at all loci in mouse *RNF43* (26 loci) and *GNAS* (125 loci)(Fig. 2A, Supplementary Tables 2, 3). Notably, HFD-induced obesity induced significant hypermethylation in three loci (#13, #15 and #25) (HFD vs. normal-fat diet [NFD] mice: 0.5 and 0.4, *p* = 0.03; 0.65 and 0.60, *p* = 0.04; 0.5 and 0.4, *p* = 0.02 for #13, #15, and #25 loci, respectively, *n* = 7 for HFD, *n* = 5 for NFD, two-sided Student’s *t*-test) (Fig. 2B). However, no significant differences were observed between HFD- and NFD-fed mice at other loci (Fig. 2 and Supplementary Fig. 2A). Further analysis of *RNF43* and *GNAS* methylation status in both normal human pancreas and early-stage PC from publicly available database revealed no significant differences (data not shown).

## Discussions

In DIO mice, abnormal methylation was observed in three loci in mouse *RNF43* of pancreatic exocrine cells, contrasting with unaltered CpG islands in *KRAS*, *CDKN2A*, *TP53*, *SMAD4* and *GNAS*. Obesity alone was unlikely to induce aberrant methylation in these genes; therefore, the prominent contribution of these genes in PC arose from genetic alterations: mutations/deletions.

Despite reports of decreased *CDKN2A* levels owing to the hypermethylation of its promoter in approximately 15% of PC cases [23], our results revealed no influence of HFD on *CDKN2A* methylation. Obesity may not have induced *CDKN2A* hypermethylation at the presymptomatic stage but induced aberrant methylation in later PC development.

We identified significant HFD-induced hypermethylation at three loci (#13, #15 and #25) in *RNF43* owing to HFD-induced obesity (Fig. 2). RNF43 plays a crucial role in the Wnt signaling pathway by ubiquitinating its cognate receptor (Frizzeled, FZD), inducing endocytosis and degradation of FZD, and subsequent loss of the Wnt signal (Fig. 2C). Simultaneously, the canonical Wnt/β-catenin pathway regulates *RNF* expression; this forms a negative feedback loop within the Wnt signaling pathway, with the loss of *RNF* expression causing abnormal augmentation of Wnt signaling (Fig. 2) [26]. The Wnt signaling pathway exhibits frequent, widespread alterations in cancer biology [27, 28]. Multiple Wnt-responsive elements (WREs) in mouse *RNF43* were located in introns #2 and #3 (Fig. 2A). Three methylation loci (#13, #15 and #25) were also located in introns #2 and #3, adjacent to WREs (Fig. 2A), potentially linking these loci with Wnt-mediated *RNF43* gene regulation. Previous investigations have linked reduced *RNF43* expression in tumors with increased cell proliferation and invasiveness, and poor survival [26]. Notably, human *RNF43* also contained WREs in intron #2, mirroring findings in the mouse genome.

We examined the methylation status of six genes in pancreatic exocrine cells of DIO mice, assuming their significant roles in PC development. Notably, the methylation levels of *KRAS*, *CDKN2A*, *TP53*, *SMAD4* and *GNAS* remained unaffected by HFD-induced obesity, suggesting that the epigenetic effects of obesity may not converge onto identical genes undergoing mutations/deletions during PC development. In a previous study, we identified over 300 DMRs in DIO mice, proposing three pathways for PC development: (i) cell hypertrophy (involving PLC, PKC, SMAD2/3 and TRKA); (ii) metabolic control (involving CREB and AMPK); and (iii) potassium regulation (involving K^+^-channel). The epigenetic effects of obesity on PC may preferentially involve genes in these pathways rather than those well-known for mutations/deletions. These findings provide key molecular insights into PC pathogenesis and potential biomarkers development through obesity-induced epigenetics.

## Limitations

The current study has several limitations. Notably, metabolic differences between mouse and human pancreatic tissues could lead to variations in obesity-induced epigenetic effects. Furthermore, there is a need to increase the sample size to strengthen our findings. The expression level of *RNF43* in obese mice was not evaluated, indicating a gap that necessitates future research to explore the correlation between DNA methylation levels at three loci, *RNF43* expression levels, and subsequent abnormal augmentation of Wnt signaling. Besides DNA methylation, epigenetic modifications involve histone modifications (*e.g.*, acetylation and deacetylation of histones) and chromatin accessibility [29, 30]. These modifications result from three biochemical reactions, including writers (adding chemical groups to DNA or histones), erasers (removing epigenetic modifications), and readers (recognizing specific epigenetic marks). Investigating how obesity-induced epigenetic changes may influence histone modifications and chromatin accessibility, and the mechanisms of these epigenetic modifications through biochemical reactions, remains an area for future exploration.

## Materials and methods

### Mice and diets

To minimize confounding risk factors associated with human PC, particularly sex, we exclusively examined female C57BL/6J mice (Charles River Japan, Tokyo). Mice were housed separately and given ad libitum access to HFD or NFD from 5 to 60 weeks of age (tissue collection). The HFD group (*n* = 7) received feed containing fat equivalent to 60% of the total calories (D12492, Research Diets Inc., New Brunswick, NJ). The NFD group (*n* = 5) received feed containing fat equivalent to 10% of the total calories (D12450J Research Diets Inc).

### Mouse pancreatic exocrine cells cultures

All procedures for the experiments using mice were approved by the Animal Committee of Saitama Medical University (protocol:2499). All methods were performed following experimental procedures mirroring those described in our previous study [25]. Briefly, mice were euthanized at approximately 60-week old with CO_2_ gas, and pancreatic tissues were excised. The tissues were minced, digested, and triturated in Hanks’ balanced salt solution (HBSS) containing 5% fetal bovine serum (FBS), 0.25 mg/ml trypsin inhibitor and 25 ng/ml epidermal growth factor (EGF) (Corning Life Sciences, Tewksbury, MA). Isolated pancreatic cells were passed through the mesh, rinsed with the medium, and plated.

### DNA methylation analysis

Methylation analyses followed the protocols outlined in our previous study (Araki 2023). Briefly, cultured pancreatic exocrine cells were recovered at ∼ 4 days in vitro and genomic DNA was prepared; genomic DNA (∼ 1 µg) was bisulfite-treated using a DNA methylation kit (Takara Bio). We analyzed methylation using an Infinium Mouse Methylation BeadChip array, validated to contain 280,754 CpG sites. Statistical analyses were conducted using Python (v3.6) and GenomeStudio Methylation Module (v1.8) available in GenomeStudio (v2011.1), with individual probes filtered based on mean q < 0.05 (FDR = 0.1, Abs(delta) > 0.2 [*i.e.*, > 20% change in β-value]). CpG site gene information for mouse *KRAS*, *CDKN2A*, *TP53* and *SMAD4* is shown in Supplementary Tables 1 and Fig. 2. Human PC stage IB methylation data (*n* = 3) were extracted from the National Cancer Institute portal (GDC portal, https://portal.gdc.cancer.gov/). Stage IB represents PC with tumor sizes ranging between 2- and 4-cm (https://www.cancer.gov/). The database lacks data on earlier PC stages, rendering Stage IB the earliest available. Probe information and the signal data were converted and imported into GenomeStudio.

### Electronic supplementary material

Below is the link to the electronic supplementary material.

Supplementary Material 1

## Data Availability

Data availability: Our DNA methylation data have been submitted to the NCBI Gene Expression Omnibus under accession number GSE214033.

## Abbreviations

CDKN2A: p16/cyclin dependent kinase inhibitor 2 A
GNAS: Guanine nucleotide binding protein, α subunit
