# Supplementary material for: Selective epigenetic alterations in RNF43 in pancreatic exocrine cells from high-fat-diet-induced obese mice; implications for pancreatic cancer
Source: BMC Res Notes. 2024 Apr 15;17:106. doi: 10.1186/s13104-024-06757-0 (PMC11020883; doi:10.1186/s13104-024-06757-0)
Supplement: Supplementary file 1 — Supplementary Material 1 [file 13104_2024_6757_MOESM1_ESM.pdf]

Supplementary material for

Selective epigenetic alteratons in RNF43 in pancreatic exocrine cells from high-fat-diet-induced obese mice; implications for pancreatic cancer.

Tomoyuki Araki[1], Naofumi Miwa[2]

[1]Department of Biochemistry, School of Medicine, Saitama Medical University, 38 Moro-hongo, Moroyama, Iruma-gun, Saitama 350-0495, Japan

[2]Department of Physiology, School of Medicine, Saitama Medical University, 38 Moro-hongo, Moroyama, Iruma-gun, Saitama 350-0495, Japan.

Supplementary Material

Supplementary material includes Supplemental Figures S1 and S2, and Supplementary Tables S1-S3.

Supplementary Figure S1 Averaged β-values of all CpGs in GNAS in HFD- and NFD-mouse pancreatic cells.Methylation levels in all CpGs (CpG#1-#125) were compared between HFD-fed-(n=7) and NFD-fed mice (n=5).

Supplementary Figure S2 Averaged β -values of all CpGs in RNF43 in HFD- and NFD-mouse pancreatic cells. Methylation levels in all CpGs (CpG#1-#26) were compared between HFD-fed-(n=7) and NFD-fed mice (n=5).

Supplementary Table S1 Information on CpG loci in mouse KRAS, p16CDKN2A, p53, and Smad4.

Supplementary Table S2 Information on CpG loci in mouse GNAS.

Supplementary Table S3 Information on CpG loci in mouse RNF43.

GNAS

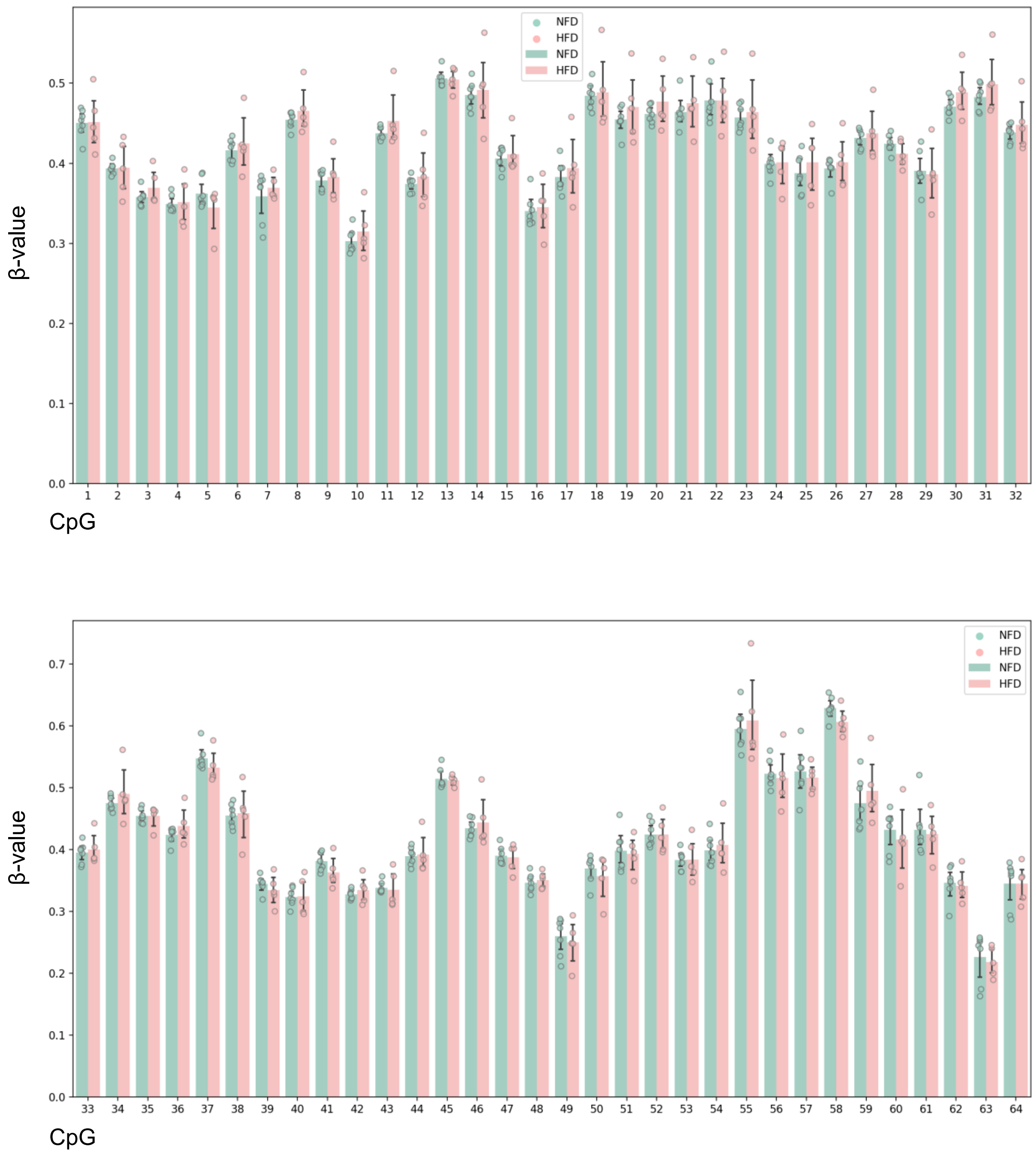

Supplementary Figure S1 (continue on next page)

GNAS

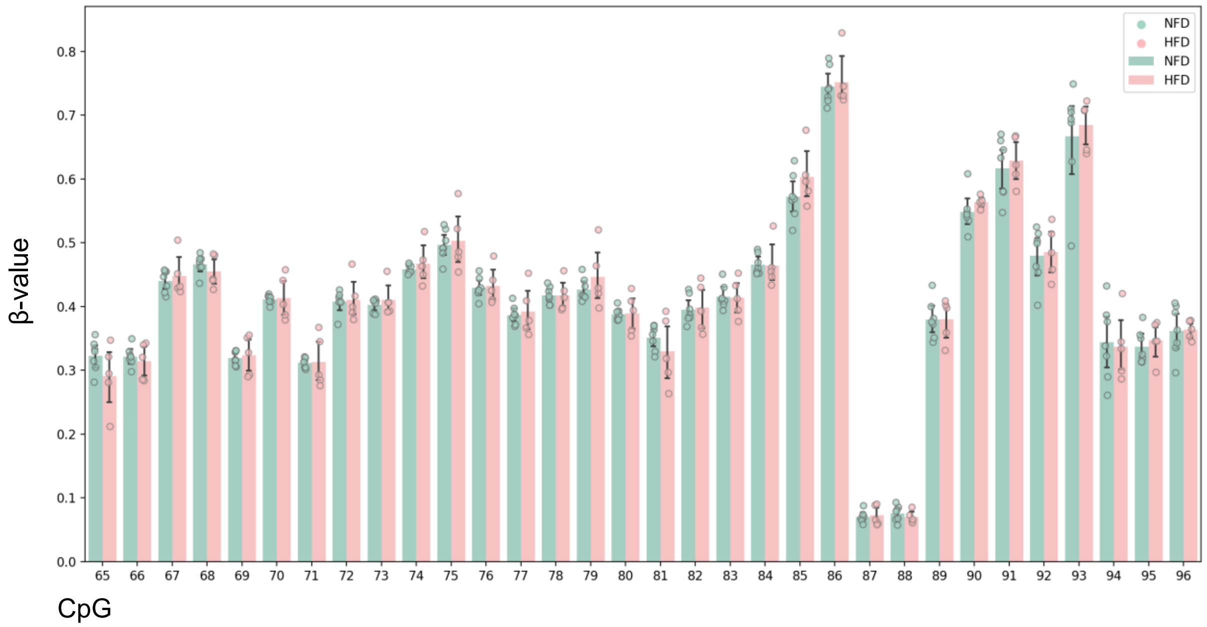

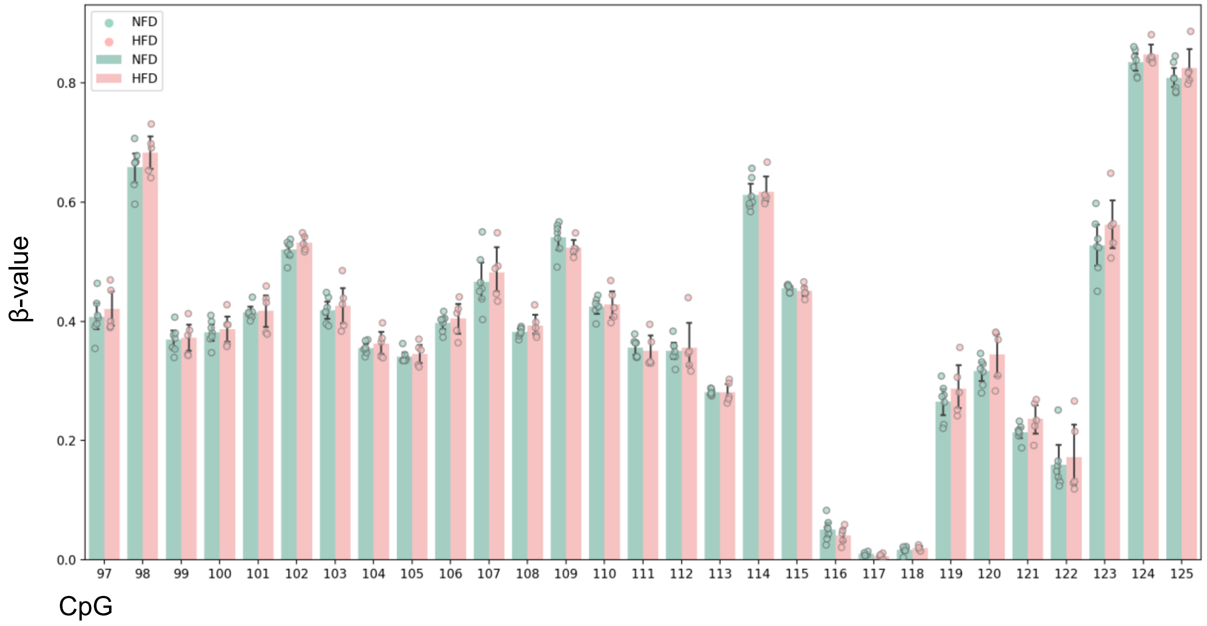

Supplementary Figure S1 (continued)

RNF43

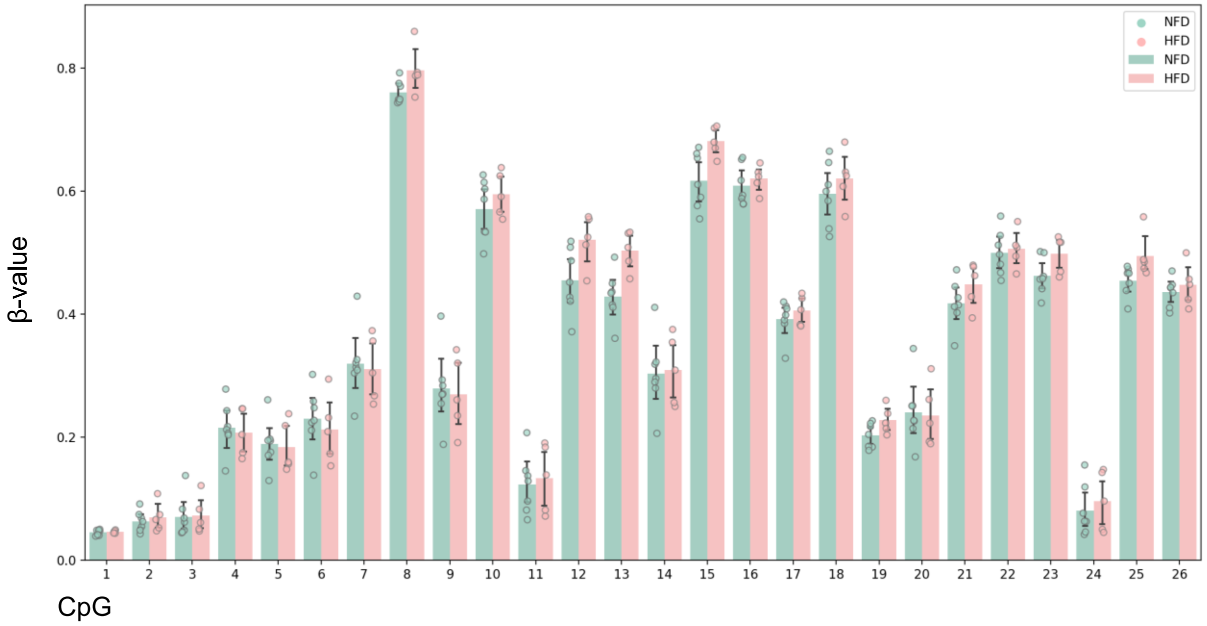

Supplementary Figure S2

	NUMBER	NAME	CHR	MAPINFO	N_SHELF	N_SHORE	CPG_ISLAND	S_SHORE	S_SHELF
KRAS	1	cg44142609	6	145216795					
	2	cg44142772	6	145244183					
	3	cg44142826	6	145249408			chr6:145249372-145250626		
	4	cg44143025	6	145250766				chr6:145249372-145250626	
	5	cg44143027	6	145250819				chr6:145249372-145250626	
	6	cg44143039	6	145251155				chr6:145249372-145250626	
CDKN2A	1	cg41120741	4	89276506		chr4:89276672-89276968			
	2	cg41120748	4	89276697			chr4:89276672-89276968		
	3	cg41120749	4	89276718			chr4:89276672-89276968		
	4	cg41120757	4	89276801			chr4:89276672-89276968		
	5	cg41120791	4	89278644				chr4:89276672-89276968	
	6	cg41120828	4	89282079					
	7	cg41120837	4	89282272					
	8	cg41120883	4	89285746					
	9	cg41120887	4	89286197					
	10	cg41120900	4	89289131					
	11	cg41120922	4	89291283	chr4:89294132-89295553				
	12	cg41120979	4	89294409			chr4:89294132-89295553		
	13	cg41120996	4	89294602			chr4:89294132-89295553		
TRP53	1	cg29707076	11	69579236	chr11:69581407-69581695		chr11:69579019-69579349		
	2	cg29707088	11	69579338	chr11:69581407-69581695		chr11:69579019-69579349		
	3	cg29707089	11	69579348	chr11:69581407-69581695		chr11:69579019-69579349	chr11:69579019-69579349	
	4	cg29707115	11	69580253		chr11:69581407-69581695		chr11:69579019-69579349	
	5	cg29707116	11	69580279		chr11:69581407-69581695		chr11:69579019-69579349	
	6	cg29707120	11	69580371		chr11:69581407-69581695		chr11:69579019-69579349	
	7	cg29707157	11	69581225		chr11:69581407-69581695		chr11:69579019-69579349	
	8	cg29707204	11	69582412				chr11:69581407-69581695	chr11:69579019-69579349
	9	cg29707207	11	69582565				chr11:69581407-69581695	chr11:69579019-69579349
	10	cg29707247	11	69584564					chr11:69581407-69581695
	11	cg29707249	11	69584734					chr11:69581407-69581695
	12	cg29707251	11	69584918					chr11:69581407-69581695
	13	cg29707292	11	69587631					
	14	cg29707360	11	69590151					
SMAD4	1	cg35911215	18	73650434					
	2	cg35911240	18	73653471					
	3	cg35911349	18	73665542					
	4	cg35911366	18	73666767					
	5	cg35911369	18	73667179					
	6	cg35911449	18	73677872					
	7	cg35911454	18	73678542					
	8	cg35911580	18	73697121					
	9	cg35911610	18	73699634	chr18:73703101-73704325				
	10	cg35911618	18	73700485	chr18:73703101-73704325				
	11	cg35911622	18	73700931	chr18:73703101-73704325				
	12	cg35911807	18	73704058			chr18:73703101-73704325		
	13	cg35911812	18	73704139			chr18:73703101-73704325		
	14	cg35911827	18	73704455				chr18:73703101-73704325	

Supplementary Table S1

	NUMBER	NAME	CHR	MAPINFO	N_SHELF	N_SHORE	CPG_ISLAND	S_SHORE	S_SHELF
GNAS	1	cg39382165	2	174283351	chr2:174286011-174286221	chr2:174284647-174285390			
	2	cg39382172	2	174283595	chr2:174286011-174286221	chr2:174284647-174285390			
	3	cg39382173	2	174283626	chr2:174286011-174286221	chr2:174284647-174285390			
	4	cg39382174	2	174283653	chr2:174286011-174286221	chr2:174284647-174285390			
	5	cg39382178	2	174283765	chr2:174286011-174286221	chr2:174284647-174285390			
	6	cg39382179	2	174283902	chr2:174286011-174286221	chr2:174284647-174285390			
	7	cg39382183	2	174283967	chr2:174286011-174286221	chr2:174284647-174285390			
	8	cg39382186	2	174284093		chr2:174284647-174285390			
	9	cg39382200	2	174284343		chr2:174284647-174285390			
	10	cg39382234	2	174284886		chr2:174286011-174286221	chr2:174284647-174285390		
	11	cg39382238	2	174284971		chr2:174286011-174286221	chr2:174284647-174285390		
	12	cg39382283	2	174285561		chr2:174286011-174286221		chr2:174284647-174285390	
	13	cg39382290	2	174285711		chr2:174286011-174286221		chr2:174284647-174285390	
	14	cg39382292	2	174285728		chr2:174286011-174286221		chr2:174284647-174285390	
	15	cg39382293	2	174285742		chr2:174286011-174286221		chr2:174284647-174285390	
	16	cg39382297	2	174285861		chr2:174286011-174286221		chr2:174284647-174285390	
	17	cg39382298	2	174285893		chr2:174286011-174286221		chr2:174284647-174285390	
	18	cg39382299	2	174285908		chr2:174286011-174286221		chr2:174284647-174285390	
	19	cg39382300	2	174285922		chr2:174286011-174286221		chr2:174284647-174285390	
	20	cg39382301	2	174285938		chr2:174286011-174286221		chr2:174284647-174285390	
	21	cg39382302	2	174285962		chr2:174286011-174286221		chr2:174284647-174285390	
	22	cg39382303	2	174285977		chr2:174286011-174286221		chr2:174284647-174285390	
	23	cg39382306	2	174286029			chr2:174286011-174286221	chr2:174284647-174285390	
	24	cg39382326	2	174286336				chr2:174284647-174285390	
	25	cg39382336	2	174286537				chr2:174284647-174285390	
	26	cg39382350	2	174286894				chr2:174284647-174285390	
	27	cg39382356	2	174287059				chr2:174284647-174285390	
	28	cg39382357	2	174287069				chr2:174284647-174285390	
	29	cg39382366	2	174287245				chr2:174284647-174285390	
	30	cg39382384	2	174287998				chr2:174286011-174286221	chr2:174284647-174285390
	31	cg39382449	2	174294260	chr2:174297262-174297532				
	32	cg39382450	2	174294283	chr2:174297262-174297532				
	33	cg39382460	2	174294755	chr2:174297262-174297532				
	34	cg39382473	2	174295221	chr2:174297262-174297532				
	35	cg39382475	2	174295314	chr2:174298463-174300644	chr2:174297262-174297532			
	36	cg39382491	2	174295512	chr2:174298463-174300644	chr2:174297262-174297532			
	37	cg39382496	2	174295627	chr2:174298463-174300644	chr2:174297262-174297532			
	38	cg39382499	2	174295728	chr2:174298463-174300644	chr2:174297262-174297532			
	39	cg39382503	2	174295815	chr2:174298463-174300644	chr2:174297262-174297532			
	40	cg39382505	2	174295848	chr2:174298463-174300644	chr2:174297262-174297532			
	41	cg39382508	2	174295954	chr2:174298463-174300644	chr2:174297262-174297532			
	42	cg39382521	2	174296217	chr2:174298463-174300644	chr2:174297262-174297532			
	43	cg39382522	2	174296229	chr2:174298463-174300644	chr2:174297262-174297532			
	44	cg39382531	2	174296349	chr2:174298463-174300644	chr2:174297262-174297532			
	45	cg39382538	2	174296509		chr2:174297262-174297532			
	46	cg39382542	2	174296681		chr2:174297262-174297532			
	47	cg39382543	2	174296763	chr2:174300746-174300959	chr2:174297262-174297532			
	48	cg39382544	2	174296821	chr2:174300746-174300959	chr2:174297262-174297532			
	49	cg39382556	2	174297275	chr2:174300746-174300959	chr2:174298463-174300644	chr2:174297262-174297532		
	50	cg39382572	2	174297476	chr2:174300746-174300959	chr2:174298463-174300644	chr2:174297262-174297532		
	51	cg39382578	2	174297531	chr2:174300746-174300959	chr2:174298463-174300644	chr2:174297262-174297532	chr2:174297262-174297532	
	52	cg39382579	2	174297552	chr2:174300746-174300959	chr2:174298463-174300644		chr2:174297262-174297532	
	53	cg39382580	2	174297575	chr2:174300746-174300959	chr2:174298463-174300644		chr2:174297262-174297532	
	54	cg39382581	2	174297611	chr2:174300746-174300959	chr2:174298463-174300644		chr2:174297262-174297532	
	55	cg39382584	2	174297823	chr2:174300746-174300959	chr2:174298463-174300644		chr2:174297262-174297532	
	56	cg39382585	2	174297850	chr2:174300746-174300959	chr2:174298463-174300644		chr2:174297262-174297532	
	57	cg39382586	2	174297856	chr2:174300746-174300959	chr2:174298463-174300644		chr2:174297262-174297532	
	58	cg39382587	2	174297885	chr2:174300746-174300959	chr2:174298463-174300644		chr2:174297262-174297532	
	59	cg39382588	2	174297909	chr2:174300746-174300959	chr2:174298463-174300644		chr2:174297262-174297532	
	60	cg39382600	2	174298056	chr2:174300746-174300959	chr2:174298463-174300644		chr2:174297262-174297532	
	61	cg39382603	2	174298166	chr2:174300746-174300959	chr2:174298463-174300644		chr2:174297262-174297532	
	62	cg39382604	2	174298185	chr2:174300746-174300959	chr2:174298463-174300644		chr2:174297262-174297532	
	63	cg39382609	2	174298371	chr2:174300746-174300959	chr2:174298463-174300644		chr2:174297262-174297532	
	64	cg39382614	2	174298473	chr2:174300746-174300959		chr2:174298463-174300644	chr2:174297262-174297532	
	65	cg39382619	2	174298572	chr2:174300746-174300959		chr2:174298463-174300644	chr2:174297262-174297532	

Supplementary Table S2 (continue on next page)

	NUMBER	NAME	CHR	MAPINFO	N_SHELF	N_SHORE	CPG_ISLAND	S_SHORE	S_SHELF
GNAS	66	cg39382641	2	174298769		chr2:174300746-174300959	chr2:174298463-174300644	chr2:174297262-174297532	
	67	cg39382642	2	174298821		chr2:174300746-174300959	chr2:174298463-174300644	chr2:174297262-174297532	
	68	cg39382645	2	174298833		chr2:174300746-174300959	chr2:174298463-174300644	chr2:174297262-174297532	
	69	cg39382657	2	174299016		chr2:174300746-174300959	chr2:174298463-174300644	chr2:174297262-174297532	
	70	cg39382665	2	174299151		chr2:174300746-174300959	chr2:174298463-174300644	chr2:174297262-174297532	
	71	cg39382666	2	174299168		chr2:174300746-174300959	chr2:174298463-174300644	chr2:174297262-174297532	
	72	cg39382691	2	174299514		chr2:174300746-174300959	chr2:174298463-174300644	chr2:174297262-174297532	
	73	cg39382741	2	174300142		chr2:174300746-174300959	chr2:174298463-174300644		chr2:174297262-174297532
	74	cg39382744	2	174300224		chr2:174300746-174300959	chr2:174298463-174300644		chr2:174297262-174297532
	75	cg39382745	2	174300253		chr2:174300746-174300959	chr2:174298463-174300644		chr2:174297262-174297532
	76	cg39382752	2	174300328		chr2:174300746-174300959	chr2:174298463-174300644		chr2:174297262-174297532
	77	cg39382766	2	174300556		chr2:174300746-174300959	chr2:174298463-174300644		chr2:174297262-174297532
	78	cg39382778	2	174300643		chr2:174300746-174300959	chr2:174298463-174300644	chr2:174298463-174300644	chr2:174297262-174297532
	79	cg39382779	2	174300693		chr2:174300746-174300959		chr2:174298463-174300644	chr2:174297262-174297532
	80	cg39382780	2	174300747			chr2:174300746-174300959	chr2:174298463-174300644	chr2:174297262-174297532
	81	cg39382783	2	174300799			chr2:174300746-174300959	chr2:174298463-174300644	chr2:174297262-174297532
	82	cg39382786	2	174300823			chr2:174300746-174300959	chr2:174298463-174300644	chr2:174297262-174297532
	83	cg39382787	2	174300868			chr2:174300746-174300959	chr2:174298463-174300644	chr2:174297262-174297532
	84	cg39382800	2	174301062				chr2:174298463-174300644	chr2:174297262-174297532
	85	cg39382826	2	174303019					chr2:174298463-174300644
	86	cg39382843	2	174306581					
	87	cg39382878	2	174311110					
	88	cg39382884	2	174312298					
	89	cg39382954	2	174319752					
	90	cg39382996	2	174325146	chr2:174327293-174331145				
	91	cg39382999	2	174325458		chr2:174327293-174331145			
	92	cg39383000	2	174325522		chr2:174327293-174331145			
	93	cg39383004	2	174325935		chr2:174327293-174331145			
	94	cg39383008	2	174326120		chr2:174327293-174331145			
	95	cg39383009	2	174326274		chr2:174327293-174331145			
	96	cg39383010	2	174326379		chr2:174327293-174331145			
	97	cg39383011	2	174326399		chr2:174327293-174331145			
	98	cg39383014	2	174326491		chr2:174327293-174331145			
	99	cg39383016	2	174326630		chr2:174327293-174331145			
	100	cg39383017	2	174326724		chr2:174327293-174331145			
	101	cg39383019	2	174326755		chr2:174327293-174331145			
	102	cg39383021	2	174326855		chr2:174327293-174331145			
	103	cg39383022	2	174326886		chr2:174327293-174331145			
	104	cg39383023	2	174326931		chr2:174327293-174331145			
	105	cg39383024	2	174326955		chr2:174327293-174331145			
	106	cg39383026	2	174327011		chr2:174327293-174331145			
	107	cg39383027	2	174327029		chr2:174327293-174331145			
	108	cg39383037	2	174327240		chr2:174327293-174331145			
	109	cg39383040	2	174327307			chr2:174327293-174331145		
	110	cg39383056	2	174327442			chr2:174327293-174331145		
	111	cg39383102	2	174327968			chr2:174327293-174331145		
	112	cg39383111	2	174328269			chr2:174327293-174331145		
	113	cg39383113	2	174328354			chr2:174327293-174331145		
	114	cg39383131	2	174328583			chr2:174327293-174331145		
	115	cg39383182	2	174329029			chr2:174327293-174331145		
	116	cg39383319	2	174330373			chr2:174327293-174331145		
	117	cg39383355	2	174330642			chr2:174327293-174331145		
	118	cg39383401	2	174331237				chr2:174327293-174331145	
	119	cg39383432	2	174334190					chr2:174327293-174331145
	120	cg39383438	2	174334592					chr2:174327293-174331145
	121	cg39383449	2	174335073					chr2:174327293-174331145
	122	cg39383466	2	174336185					
	123	cg39383545	2	174342037					
	124	cg39383618	2	174346443					
	125	cg39383619	2	174346491					

Supplementary table S2 (continued)

	NUMBER	NAME	CHR	MAPINFO
RNF43	1	cg29893609	11	87662645
	2	cg29893615	11	87662988
	3	cg29893618	11	87663039
	4	cg29893644	11	87664460
	5	cg29893646	11	87664529
	6	cg29893662	11	87665372
	7	cg29893802	11	87682978
	8	cg29893832	11	87686190
	9	cg29893857	11	87687794
	10	cg29893866	11	87688269
	11	cg29893883	11	87689569
	12	cg29893919	11	87691869
	13	cg29893927	11	87692386
	14	cg29893983	11	87697212
	15	cg29894002	11	87697987
	16	cg29894016	11	87699836
	17	cg29894017	11	87699857
	18	cg29894041	11	87701723
	19	cg29894047	11	87702041
	20	cg29894056	11	87702394
	21	cg29894095	11	87705080
	22	cg29894122	11	87707546
	23	cg29894142	11	87709181
	24	cg29894160	11	87710117
	25	cg29894301	11	87720518
	26	cg29894375	11	87726994

	CHR	MAPINFO
**WRE1**	11	87558680
	11	87572744
	11	87573283
	11	87578951
	11	87581851
	11	87587910
	11	87603458
	11	87605819

	CHR	MAPINFO
**WRE2**	11	87563495
	11	87565945
	11	87580380
	11	87590079
	11	87592983
	11	87611912
	11	87613120
	11	87613710
	11	87616154

Supplementary Table S3
